# Developments in Combining Targeted Radionuclide Therapies and Immunotherapies for Cancer Treatment

**DOI:** 10.3390/pharmaceutics15010128

**Published:** 2022-12-30

**Authors:** Caroline P. Kerr, Joseph J. Grudzinski, Thanh Phuong Nguyen, Reinier Hernandez, Jamey P. Weichert, Zachary S. Morris

**Affiliations:** 1 Department of Radiology, School of Medicine and Public Health, University of Wisconsin-Madison, Madison, WI 53705, USA; 2 Department of Human Oncology, School of Medicine and Public Health, University of Wisconsin-Madison, Madison, WI 53705, USA; 3 Department of Medical Physics, School of Medicine and Public Health, University of Wisconsin-Madison, Madison, WI 53705, USA

**Keywords:** targeted radionuclide therapy (TRT), anti-tumor immunity, external beam radiation therapy (EBRT), tumor microenvironment (TME)

## Abstract

Targeted radionuclide therapy (TRT) and immunotherapy are rapidly growing classes of cancer treatments. Basic, translational, and clinical research are now investigating therapeutic combinations of these agents. In comparison to external beam radiation therapy (EBRT), TRT has the unique advantage of treating all disease sites following intravenous injection and selective tumor uptake and retention—a particularly beneficial property in metastatic disease settings. The therapeutic value of combining radiation therapy with immune checkpoint blockade to treat metastases has been demonstrated in preclinical studies, whereas results of clinical studies have been mixed. Several clinical trials combining TRT and immune checkpoint blockade have been initiated based on preclinical studies combining these with EBRT and/or TRT. Despite the interest in translation of TRT and immunotherapy combinations, many questions remain surrounding the mechanisms of interaction and the optimal approach to clinical implementation of these combinations. This review highlights the mechanisms of interaction between anti-tumor immunity and radiation therapy and the status of basic and translational research and clinical trials investigating combinations of TRT and immunotherapies.

## 1. Introduction

Metastatic disease carries a poor prognosis for cancer patients, and little progress has been made to change this reality, despite advances in individual applications of immunotherapy, chemotherapy, and radiation therapy. It is estimated that 90% of patients who die from cancer die from metastases [1]. Systemic therapy is critical for metastatic disease, and combining targeted radionuclide therapy (TRT; also referred to as radiopharmaceutical therapy) and immunotherapy has the potential to achieve improved tumor response compared to monotherapies. The promise of TRT in treatment of metastatic disease and an evolving understanding of the immunogenicity of radiation has led to preclinical and clinical investigations of combination TRT and immunotherapy treatments, which we aim to explore here.

It has been known for over a century that radiation interacts with the immune system [2]. Radiation therapy is classically thought to exert its damaging effects through DNA damage resulting in mitotic catastrophe in tumor cells [3]. This radiation-induced tumor cell death has been shown to be immunogenic in nature [4,5]. Immunostimulatory mechanisms of interaction between external beam radiation therapy (EBRT) and the tumor microenvironment (TME) include immunogenic cell death (ICD), release of pro-inflammatory cytokines, and upregulation of tumor antigen-presenting complexes [6]. Extracellular ATP release post EBRT-induced ICD, calreticulin surface exposure, and High Mobility Group Box 1 (HMGB1) release are all implicated in dendritic cell and CD8^+^ effector T cell activation [7]. Several pro-inflammatory cytokines are also released in response to EBRT, including IL-1β, TNF-α, and IL-12, further favoring anti-tumor immune responses in the TME [4,8].

In several preclinical studies, combination of EBRT and immune checkpoint inhibitors (ICI) has resulted in synergistic tumor responses, including tumor elimination [9,10]. These responses are aided by the immune activation mechanisms above, whereby radiation sensitizes tumors to antibody blockade of inhibitory immune checkpoints (e.g., programmed cell death protein 1 (PD-1) and its ligand, PD-L1; cytotoxic T lymphocyte antigen 4 (CTLA-4)). Clinically results have been mixed with at least one study suggesting benefit from combining ICI therapy and EBRT. Theelen et al. randomized 76 metastatic non-small cell lung cancer patients to receive combination radiotherapy (8 Gy × 3) and pembrolizumab or pembrolizumab alone in a phase II trial (PEMBRO-RT). A doubling of overall response rate at 12 weeks was observed in the combination therapy arm compared to monotherapy, but it did not reach statistical significance (*p* = 0.07). The largest benefit of combination therapy was observed in the subgroup with PD-L1 negative tumors, highlighting the potential of radiotherapy to enhance responses to ICI in immunologically cold (i.e., low tumor mutational burden, low baseline immune cell infiltrate) tumors [11].

Abscopal effects are sometimes observed where irradiation leads to partial or complete responses at unirradiated sites in patients with metastatic disease. These abscopal effects do not typically result in a sustained systemic anti-tumor response following EBRT monotherapy [4,8,12,13,14]. A systematic review of abscopal effect case reports by Abuodeh and others identified 46 cases where reported median radiation dose was 31 Gy, median documented time to observation of the effect was two months, and median documented time to progress at the site of abscopal effect was six months. The rarity of the abscopal responses suggests that there is high threshold to the effect, posing a major barrier to reliable clinical translation of this effect as a strategy for activating effective systemic anti-tumor immunity [14].

One reason for the rarity of the abscopal effect may be that immunosuppressive cells and microenvironments in distant non-irradiated tumor sites can inhibit the propagation of a systemic anti-tumor response from a focally irradiated tumor. Illustratively, we have shown that the in situ vaccine effect of EBRT was enhanced when combined with tumor-targeted hu14.18-IL2 immunocytokine injections [15], yet in the presence of multiple tumors, the same combination therapy targeted to a single tumor was ineffective [16]. This phenomenon, observed only with multiple tumors of the same type, was coined “concomitant immune tolerance” (CIT) [16]. CIT may therefore be a critical barrier to the application of combination EBRT-immunotherapy in metastatic disease settings. Radiating all tumor sites overcomes CIT and restores in situ vaccine efficacy [16]. Similarly, Yu et al. showed loss of response to anti-PD-L1 therapy of a subcutaneous MC38 colon carcinoma tumor in the presence of a liver metastasis and observed that liver-directed radiotherapy restored the systemic anti-tumor immune response [17]. For most patients with metastatic disease, it is not possible to treat all tumors with EBRT without incurring systemic immunosuppression that would preclude effective combination with immunotherapy.

TRTs are a class of cancer therapy that has been demonstrated to increase the survival of patients with metastatic lymphoma, thyroid, prostate, neuroendocrine, and liver cancers [18,19,20,21,22]. Following administration to patients, commonly by intravenous injection, TRTs deliver radionuclides to tumors via a targeting vector that is selectively taken up and retained in tumor cells or the TME. Theranostics is an emerging field in oncology utilizing a TRT to image (diagnose) and deliver therapy to treat tumors [23].

TRT agents have been used for cancer treatment for close to a century, with the most well-known application being ^131^I in the treatment of thyroid cancers, changing a disease with poor prognosis to a disease with about 85% long-term survival [23]. The scope of TRT applications has broadened significantly in recent years. One catalyst for this resurgence was the 2013 US Food and Drug Administration (FDA) approval of ^223^Ra (Xofigo^®^) for treatment of prostate cancer patients with symptomatic bone metastases. The ALSYMPCA trial randomized 921 patients with histologically confirmed, metastatic castration-resistant prostate cancer (mCRPC) with two or more bone metastases to 6 cycles of Xofigo versus standard of care and demonstrated a 3.6-month improvement in overall survival [24,25]. TRT momentum further increased with the 2018 FDA approval of ^177^Lu-DOTATATE (Lutathera^®^) for neuroendocrine tumors [26] and the 2022 approval of ^177^Lu-PSMA-617 (Pluvicto^TM^) for the treatment of mCRPC following the VISION trial. This phase III trial randomized 831 patients with mCRPC to 4–6 cycles of ^177^Lu-PSMA-617 plus standard care or standard care alone, resulting in 5.3-month improvement in progression free survival and 4.0 month increase in overall survival following ^177^Lu-PSMA-617 [27].

### 1.1. TRT Radiobiology

TRT has distinct radiobiological properties from EBRT. Targeting vectors for TRT include antibodies, antibody fragments/platforms, proteins, peptides, and other small molecules [23]. TRT delivers a heterogenous dose of radiation within tumors, at continuous low absorbed dose rates (e.g., <0.5 Gy/h), and with radionuclide-dependent variable linear energy transfer (LET). Conversely, conventional EBRT is typically delivered in homogeneous doses, with low LET protons, photons, and electrons (0.2 keV/µm), and at fractionated high absorbed dose rates (e.g., 1–2 Gy/min). As a result of differing dose-rates, LET, volume of tissue irradiated, and off-target sites of dose delivery, the biological response to TRT differs from the response to EBRT [28,29].

**Figure 1:**
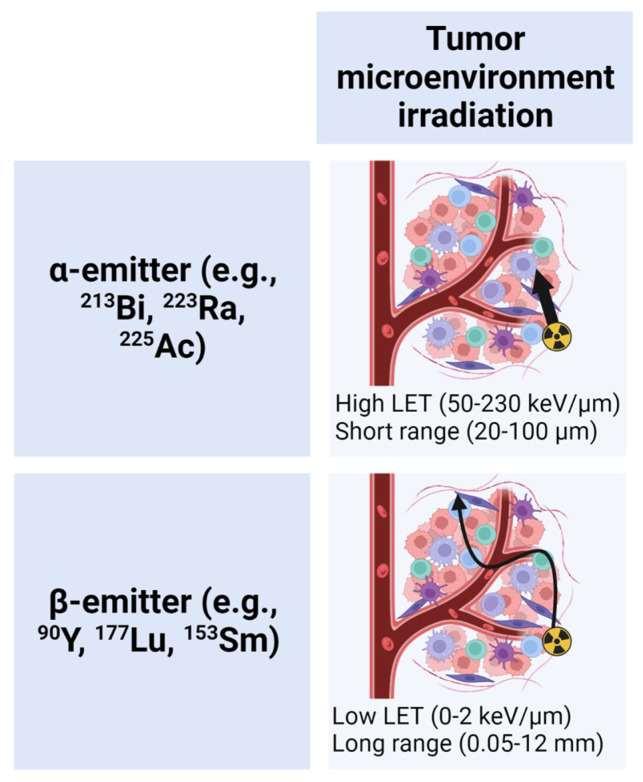
Tumor microenvironment irradiation by alpha-emitters versus beta-emitters. Physical property differences of alpha particles and beta particles lead to different tumor irradiation patterns. Alpha particles (high linear energy transfer (LET), short tissue range) traverse 1–2 cell diameters with a high density of ionization events along their track. Beta particles (low LET, long tissue range) travel millimeters in tissue with sparse ionization events, and have the potential to irradiate many structures in addition to the cell of targeted radionuclide therapy (TRT) uptake. Created with BioRender.com.

TRT radionuclide selection includes radionuclides emitting β particles, α particles, γ rays, and/or Auger electrons. Clinical applications of radionuclides are determined by the energy released by the emitted particles over unit distance (i.e., LET), their range in tissue, and their physical half-life [29]. The low LET (0–2 keV/µm) and long-range emission (0.05–12 mm) of β emitters (e.g., ^90^Y, ^177^Lu) results in irradiation of the tumor microenvironment in addition to the tumor cells with TRT uptake [29]. This allows for neighboring cells without expression of the molecular target to also be irradiated (i.e., crossfire effect), overcoming potential heterogenous target expression among cancer cells [29]. α emitters (e.g., ^223^Ra) are characterized by a short tissue range (20–100 µm) and high LET (50–230 keV/µm) [30]. High LET translates into local dose deposition with a high density of ionization events [31]. The tumor microenvironment irradiation by alpha- and beta-particle-emitting radionuclides based on their physical properties is shown in Figure 1. Auger electrons irradiate volumes with subcellular dimensions since the LET of an Auger electron is 4–26 keV/μm, and its range in tissue is less than 20 μm. Given higher LETs and short range, both α emitters and Auger electrons have greater potential to induce cell death in target cells of uptake. In order to kill a cell, 100–1000 beta particle traversals are required [32], versus 1–20 alpha particle traversals [33]. Radionuclides emitting γ rays are often used in dual therapeutic and diagnostic applications as they can be used for imaging and therapy [29].

Absorbed dose, the energy absorbed per unit mass (1 J/kg = Gy), of EBRT can predict the biological response [34]. However, the radiobiology of TRT is not as clearly understood as that of EBRT [35]. The emission properties of a radionuclide [36], the number of target cells, and characteristics of the tumor itself, including tumor radiosensitivity, perfusion, and permeability [37,38], often lead to heterogeneous TRT dose distributions. One implication of heterogeneous dose distribution is that it renders average tumor absorbed doses and whole organ absorbed doses less useful predictors of therapeutic efficacy and organ toxicity, respectively [39]. Historically, patient-specific dosimetry was not standardly used for TRT, making it very challenging to determine the biological effects of absorbed dose from TRT. The Society of Nuclear Medicine and Molecular Imaging (SNMMI) Dosimetry Task Force published a series of articles in 2021 describing the progress and challenges surrounding radiopharmaceutical dosimetry [40]. These articles emphasize the importance of patient-specific imaging-based dosimetry to guide TRT administration in clinical practice [41,42,43,44,45,46,47].

### 1.2. Immunomodulation by TRT

**Figure 2:**
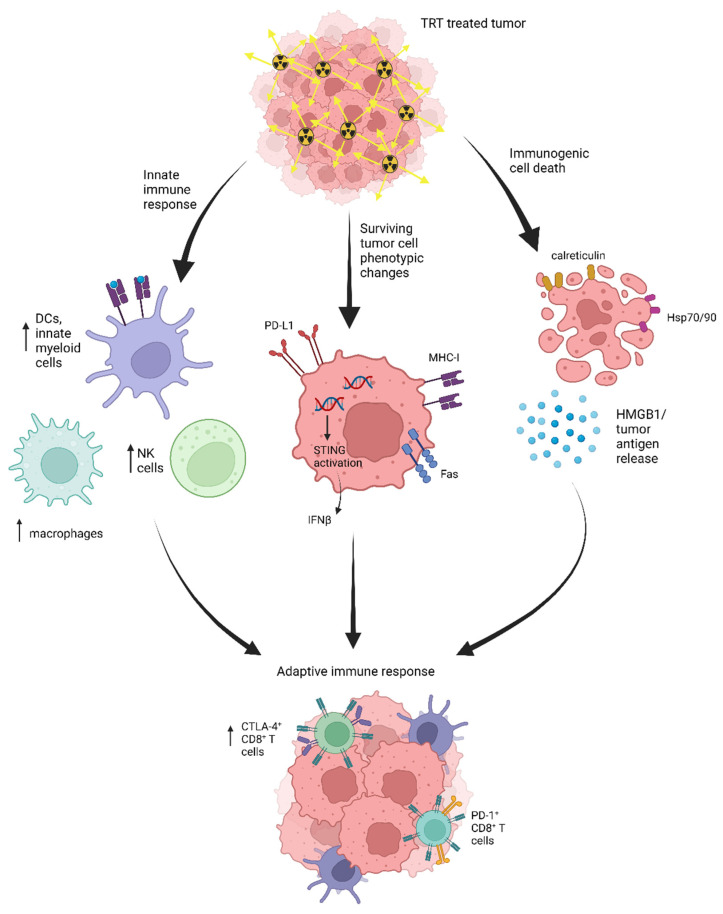
Schematic of immune activation mechanisms by targeted radionuclide therapy (TRT). Initially, tumor cells treated by TRT undergo immunogenic cell death (ICD), releasing tumor specific antigens in the process, while other surviving cells undergo phenotypic changes. These include increased PD-L1, MHC-I, and Fas expression, as well as IFNβ production through activation of the cGAS/STING pathway. Additionally, innate immune cell populations increase in the tumor microenvironment (TME), including dendritic cells (DCs), natural killer (NK) cells, macrophages, and other innate myeloid cells. Finally, a robust adaptive immune response is generated, characterized by increased CTLA-4^+^ CD8^+^ and PD-1^+^ CD8^+^ T cell populations, as well as effector memory T cell populations, and both increases and decreases in regulatory T cell populations. Created with BioRender.com.

Many of the key immunogenic effects of EBRT have been evaluated in the context of TRT in preclinical studies with various radiopharmaceuticals. These effects are summarized in Figure 2. Immunogenic cell death is induced by TRT and leads to expression of damage-associated molecular pattern molecules (DAMPs) by tumor cells. These include the expression of calreticulin and annexin A1 on cell surfaces, alongside HMGB1 and ATP release in the extracellular matrix and secretion of type I interferon (IFN-1) [48]. [^131^I]ICF01012 (a radiolabeled melanin ligand for metastatic melanoma), ^223^Ra, and ^213^Bi (an α-emitter generated from ^225^Ac/^213^Bi generator) demonstrated activation of immunogenic cell death via upregulation of calreticulin ([^131^I]ICF01012 and ^223^Ra) and HMGB1/heat shock protein 70 (Hsp70) (^213^Bi) [48,49,50].

In cells surviving TRT, several phenotypic changes have been reported. For example, upregulation of major histocompatibility complex-I (MHC-I) expression is important in antigen-specific CD8^+^ cytotoxic T lymphocyte (CTL)-mediated killing of cancer cells. Administration of 4 and 10 Gy doses of ^223^Ra (Xofigo^®^) to human breast (MDA-MB-231, ZR75–1), prostate (LNCaP, PC3), and lung carcinoma (H1703, H441) showed at least two-fold increases in human leukocyte antigens (HLA)-A, B, and C (HLA-ABC) expression, relative to mock-irradiated controls [49]. Another study demonstrated that after exposure to 25 Gy ^153^Sm-ethylenediaminetetramethylenephosphonate (Quadramet^®^), a radiopharmaceutical complex that binds avidly to hydroxyapatite in bone, the LNCaP prostate cancer cell line showed increased MHC-I expression levels on cells, with no significant change in the percentage of cells positive for MHC-I [51]. Because of increased antigen presentation, TRT-treated tumor cells were more susceptible to tumor antigen-specific cytotoxic T lymphocyte (CTL) killing in vitro in both of these studies. ^223^Ra, given at 4- or 10-Gy doses, increased the sensitivity of all previously mentioned cell lines to CTL-mediated lysis targeting the carcinoembryonic antigen (CEA; HLA-A2-restricted), mucin-1 (MUC-1; HLA-A2-restricted), and brachyury (HLA-A2/A24-restricted) tumor antigens [49]. Exposure of LNCaP to 25- or 50-Gy doses of ^153^Sm-EDTMP showed increased sensitivity to prostate-specific antigen (PSA)-specific CTL-mediated killing (10% and 80%, respectively), compared with 0 Gy [51].

Additionally, ^90^Y-NM600 (a radiolabeled tumor targeting alkylphosphocholine analog) was shown to activate stimulator of interferon genes (STING) in cells surviving radiation. Administration of 20 μCi or 100 μCi (2.5 Gy or 12 Gy tumor absorbed dose, respectively) ^90^Y-NM600 in an immunologically cold syngeneic murine model of head and neck cancer, MOC2, resulted in IFN1 activation and induction of immune susceptibility markers (*Fas, Mhc-1, Pd-l1*, death receptor 5 (*Dr5*)) that were comparable to dose equivalent EBRT (2.5 Gy, 12 Gy) treatments in vivo [52]. Additionally, STING pathway activation was necessary to the synergy between 50 μCi ^90^Y-NM600 and anti-CTLA4 in B16 melanoma, highlighting the essential role of this pathway in certain combination TRT and immunotherapy regimens [53].

Innate immune populations involved in the initial response to radiation therapy and establishing an adaptive response have been shown to be affected by TRT. Gorin et al. demonstrated that ^213^Bi treated MC38 cells increased dendritic cell (DC) maturation and activation in vitro [50]. Patel et al. noted increased myeloid cells and natural killer (NK) cells in B78 melanoma tumors one and seven days, respectively, following 50 μCi (2.5 Gy) ^90^Y-NM600 [53]. In 30 patients with mCRPC receiving ^223^Ra, peripheral blood mononuclear cell (PBMC) phenotyping over six months revealed an immunosuppressive phenotype with an increase in both myeloid-derived suppressor cells (MDSCs) and regulatory T cells (Tregs), and an overall decrease in absolute lymphocyte counts. No differences in PBMC populations were noted in subgroup analysis of patients with alkaline phosphatase response [54]. Whether these PBMC changes reflect TME immune cell populations is unknown, but this initial study suggests that it will be important to understand these immunosuppressive mechanisms and how they may affect anti-tumor immunity.

The effect of dose and emission type of TRT on the adaptive immune response over time has not been fully elucidated. Substantial variations have been reported in tumor infiltrating lymphocyte (TIL) populations following TRT. Combination ^90^Y-NM600 and anti-CTLA4 therapy increased CD8^+^ and effector memory CD8^+^ T cells in B78 melanoma tumors 25 days following 2.5 Gy TRT, but TRT monotherapy did not affect CD8^+^ populations [53]. However, in EL4 tumors, a B6-derived murine T-cell lymphoma cell line, Hernandez et al. observed increased CD8^+^ T cell populations and decreased FOXP3^+^ cells at day 6 following ^90^Y-NM600 monotherapy [55]. In the murine adenocarcinoma MC38, vaccination by subcutaneous injection of ^213^Bi-treated MC38 cells protected against future MC38 tumor engraftment. This effect was not observed in immunodeficient athymic (nude) mice, indicating T cell involvement in mediation of the anti-tumor effect [50]. Chen et al. observed greater frequencies of intratumoral CD8^+^ T cells in combination ^177^Lu TRT + anti-PD-L1 treated tumors but not monotherapy at day 4, with loss of the effect at day 14 in MC38 tumors following integrin α_v_β_3_-targeted ^177^Lu +/− anti-PD-L1 [56]. Wen et al. demonstrated upregulation of PD-L1 on CT26 and MC38 colon adenocarcinoma tumors following treatment with integrin α_v_β_3_ targeted ^177^Lu, showing a cooperative effect between TRT agents and immune checkpoint blockade [57]. Despite promising immune activation mechanisms, TRT monotherapy has had limited durability in its efficacy against solid tumors, providing rationale for the combination of TRT with immunotherapies [58].

## 2. Combination TRT and Immunotherapies

### 2.1. Preclinical Studies

Two of the earliest preclinical examples of the therapeutic interaction between TRT and immunotherapy were reported by Chakraborty et al. in 2008 [59] and Sharkey et al. in 2009 [60]. Chakraborty et al. observed significantly improved overall survival in mice bearing MC38-CEA^+^ colon tumors when treated with a ^90^Y radiolabeled anti-CEA antibody in combination with a CEA/TRICOM vaccine, as compared to TRT or immunotherapy monotherapy [59]. Sharkey et al. noted that when radiolabeled and unlabeled anti-CD20 antibodies were combined, an increased treatment response was observed in mice bearing non-Hodgkin lymphoma tumors. Although a range of unlabeled antibodies with different immunologic targets (e.g., anti-CD22, anti-CD74) and administration schedules were studied, this cooperative response was only observed with ^90^Y-veltuzumab and unlabeled veltuzumab initiated one week after TRT [60].

The most studied form of immunotherapy in combination with TRT are ICIs. A summary of preclinical studies combining TRT and ICIs is presented in Table 1.

We have observed a cooperative therapeutic interaction of low dose ^90^Y delivered with NM600 and anti-PD-L1 and anti-CTLA-4 in syngeneic murine B78 melanoma models. This ^90^Y-NM600 + dual ICI resulted in tumor regression and improved survival at approximately 2.5 Gy tumor dose [53]. Additionally, ^90^Y-NM600 at absorbed doses of 2–5 Gy stimulated expansion of T-cell receptor (TCR) clones among tumor infiltrating lymphocytes when combined with anti-CTLA-4 without increasing diversity of the TCR repertoire [53]. This enhanced clonal expansion increased response to ICIs of these immunologically cold tumors. Promotion of clonal expansion represents a distinct immunomodulatory mechanism of TRT [53], in contrast to the diversification of T cell receptors seen with EBRT [9,73]. With the same vector, we have demonstrated that moderate dose (12 Gy) ^90^Y-NM600 in combination with dual ICI prolongs survival over each monotherapy and EBRT to the primary tumor in mice bearing two MOC2 head and neck squamous cell carcinoma tumors [52]. Finally, in prostate cancer, Potluri et al. observed that using a Treg-depleting anti-CTLA-4 isotype is critical for the synergy between ^90^Y-NM600 and ICI [72]. This result highlights the importance of understanding TRT effects on TIL populations, as these immune cell populations may predict tumor response to ICIs alone and in combination with TRT.

Using a different radionuclide and vector, Chen et al. demonstrated that ^177^Lu bound to the α_v_β_3_ integrin-targeting peptide Arg-Gly-Asp (^177^Lu-RGD) improved tumor control and increased overall survival in mice bearing MC38 colon cancer tumors when combined with anti-PD-L1 [56]. Of note, the investigators also observed increased PD-L1 expression on T cells following TRT and an increase in CD8^+^ tumor-infiltrating lymphocytes following combination therapy [56]. In another preclinical study, the small molecule TRT agent ^177^Lu-LLP2A, targeting very late antigen-4 (VLA-4), when combined with anti-CTLA-4, anti-PD-1, or anti-PD-L1 resulted in improved survival of mice bearing B16F10 melanoma tumors. This survival advantage was observed in comparison to dual ICI or TRT monotherapy [61]. An additional study in B16F10-bearing mice showed improved survival of mice treated with ^131^I-ICF01012 (a radiolabeled melanin ligand) + anti-CTLA-4 over ICI therapy alone [48].

The number of TRT targeting moieties continues to grow, with peptides, small molecules, and antibodies representing the three main classes. Ferreira et al. noted a significant overall survival advantage following treatment with a ^90^Y-labeled granzyme B targeted peptide (GZP) and anti-PD-1 in two murine colon cancer tumor models [70]. Employing both cold (unlabeled) anti-PD-L1 and ^131^I-anti-PD-L1, Wen et al. observed high tumor to normal tissue ratios of ^131^I-anti-PD-L1, and a significant survival benefit of combination therapy compared to control groups in MC38 and CT26 murine colon adenocarcinoma [71].

In addition to these reports, a growing number of studies (listed in Table 1) have demonstrated similar survival advantages of both beta-emitting (Lu-177, Y-90) [57,63,67,68] and alpha-emitting (Ac-225, At-211, Pb-212, Th-227) [63,64,65,66,69] TRTs in combination with ICIs. Jiao et al. showed that treatment of Cloudman S91 melanoma with an anti-melanin antibody (h8C3) labeled with alpha-emitting ^213^Bi in combination with anti-PD-1 led to tumor regression and improved overall survival over ICI monotherapy [62]. Using the same vector, the group demonstrated the survival benefit of low dose ^177^Lu-h8C3 + anti-PD-1, but no benefit to treatment with low or high dose ^225^Ac-h8C3 +/− anti-PD-1 [63]. This study suggests differences in tumor immune response to different radioisotopes. Investigations into effects of dose, LET, and relative timing of TRT and immunotherapy administration on the TME will be critical to understand these treatment effects.

The rapidly increasing publication rate in the TRT-immunotherapy arena reflects the promise of these initial results. Taken together, this body of preclinical work suggests synergy between the therapeutic mechanisms of TRT and ICIs and provides data supporting clinical testing of such combinations.

### 2.2. Clinical Studies

On the heels of promising preclinical data regarding the therapeutic advantage of combining EBRT and immunotherapy, several clinical trials to assess the safety and efficacy of TRT-immunotherapy have been initiated. Table 2 provides a summary of combined TRT-immunotherapy clinical trials, the majority of which are ongoing. These Phase I and II trials of combination TRT-immunotherapy primarily use the FDA-approved agents ^177^Lu-DOTATATE, ^177^Lu-PSMA-617, and ^223^RaCl_2_. Highlights from reported results are described here.

As is evident from Table 2, early interest in clinical combinations of TRT and immunotherapies centered on mCRPC. In a Phase II trial of ^153^Sm-ethylene diamine tetramethylene phosphonate (^153^Sm-EDTMP; Quadramet^®^), a beta emitter that binds osteoblastic bone lesions, and PSA/TRICOM vaccine, a therapeutic mCRPC vaccine (NCT00450619) [74], 18 patients received TRT alone and 21 received combination therapy. There was no statistically significant difference in progression free survival at four months, the primary endpoint. However, combination therapy did result in four patients with PSA decline of >30%, compared to no patients in the TRT monotherapy group [74].

Another Phase II mCRPC clinical trial (NCT02463799) incorporated the immunotherapy Sipuleucel-T (Provenge^®^), an autologous cellular therapy designed to stimulate an effector T cell response against tumor cells [75]. Sipuleucel-T was demonstrated to prolong survival compared to placebo in separate phase III trials with a median benefit at least as long (or longer) than what was previously observed with chemotherapy, ^223^Ra, or other hormonal agents, leading to its 2010 FDA approval [76]. This promising novel immunotherapy was studied in combination with the calcium mimetic ^223^Ra in a Phase II clinical trial with 32 patients, where patients receiving combination therapy had longer overall and progression-free survival and were more likely to demonstrate a >50% PSA decline; however had decreased prostatic acid phosphatase (PAP)-specific peripheral T cell proliferation compared to patients receiving sipuleucel-T alone [77].

Six of the ongoing combination TRT-ICI trials are targeting patients with mCRPC—two combining ^177^Lu-PSMA-617 and pembrolizumab (anti-PD-1; NCT03805594, NCT03658447), three combining ^223^Ra with ICIs (NCT02814669, NCT03093428, NCT04071236), and one pairing PSMA-targeting ^225^Ac-J591 with pembrolizumab (NCT04946370). In addition to assessing the safety of these therapies, the collective results of these trials will begin to inform how high LET, alpha-emitting ^223^Ra and ^225^Ac differ from low LET, beta-emitting ^177^Lu in terms of enhancing response to ICIs. Two case reports described patients with neuroendocrine tumors [78] and mCRPC [79] who responded to ^213^Bi-DOTATOC and ^225^Ac-PSMA-617, respectively, following disease progression or prohibitive hematologic toxicity on corresponding beta-emitter therapy. Alpha-emitting TRT resulted in neuroendocrine tumor responses with lower hematologic toxicity than beta-emitting TRT for a neuroendocrine tumor patient with disseminated bone marrow disease, an advantage gained from the short tissue range of alpha particles [78].

Despite positive Phase III results of Xofigo^®^ monotherapy [24], early investigations of ^223^RaCl_2_ combination therapy have not demonstrated a therapeutic benefit. In an ongoing Phase II study (NCT03093428) randomizing 42 patients with mCRPC to receive ^223^RaCl_2_ +/− pembrolizumab, the combination therapy group did not have improved overall survival nor progression free survival compared to ^223^RaCl_2_ monotherapy [80]. In a phase Ib study (NCT02814669) of atezolizumab (anti-PD-L1) and ^223^RaCl_2_ in men with mCRPC having progressed on androgen pathway inhibitor treatment, combination therapy had greater toxicity than either monotherapy, without clinical benefit. Out of 44 patients enrolled, 23 (52.3%) experienced a grade 3/4 adverse event [81]. The PRINCE trial (NCT03658447) is a Phase Ib/II study evaluating the combination of ^177^Lu-PSMA-617 and pembrolizumab in mCRPC with encouraging interim results [82]. Out of 37 patients receiving combination therapy, the PSA response rate was 76% and partial response rate was 70% for patients with Response Evaluation Criteria in Solid Tumors (RECIST) measurable disease, at a median follow up of 16 months [82].

Phase I results from a trial studying combination nivolumab (anti-PD-1) and Lutathera for extensive-stage small cell lung cancer (NCT03325816) reported a tolerable toxicity profile. Of seven patients with CT-measurable disease, one patient with extensive-stage disease had a partial response, and two with pulmonary atypical carcinoid disease had stable disease for six months. Of note, the patient with extensive-stage disease with a partial response showed the highest tumor uptake of ^68^Ga-DOTATATE on PET/CT of the seven patients evaluated [83].

Impressive responses to combination peptide receptor radionuclide therapy (PRRT; ^177^Lu-DOTATATE/DOTATOC) and ICIs in ICI-refractory metastatic Merkel cell carcinoma (mMCC), a skin cancer with occasional responses to PRRT, have been described in recent case reports [84,85]. In one report, a patient with mMCC with high metastatic burden who was resistant to avelumab and eventually progressed on ipilimumab/nivolumab, had a partial response to four additional doses of ipilimumab/nivolumab in combination with two cycles of ^177^Lu-DOTATOC. This response was maintained for five months (until time of manuscript submission) [85]. In a second report, two patients with mMCC with primary resistance to avelumab received 6 cycles of ^177^Lu-DOTATATE in combination with pembrolizumab. Both patients had PET/CT demonstrated responses at all disease sites for 3.6 and 4.8 months, respectively [84]. These exciting case reports have led to the announcement of a Phase II trial investigating ^177^Lu-DOTATATE in combination with pembrolizumab in patients with MCC (NCT05583708). Additionally, survival data from the GoTHAM trial (NCT04261855) combining ^177^Lu-DOTATATE and avelumab for treatment of mMCC will provide further insight into treatment efficacy when available. Notably, despite promising preclinical results from dual immune checkpoint blockade therapies in combination with TRT, there is yet to be a randomized controlled trial investigating this combination. Collectively, these preliminary results will lay the foundation for future Phase III trials of combination TRT-immunotherapy.

## 3. Outlook

As more developments are made in the field of combination TRT-immunotherapy, several critical differences between TRT and conventional EBRT must be understood to ensure effective and timely implementation of these exciting novel therapeutic options for metastatic disease. A detailed understanding of TRT dosimetry, toxicity, and immunomodulatory effects, as well as tumor immune escape pathways will be essential to enable effective translation of these treatments. Synchronously with mechanistic studies, determination and standardization of multidisciplinary approaches to implement these therapies will be vital to effective application of TRT-immunotherapy combinations [23]. Potential roadblocks to clinical adoption include poor understanding of radiobiology and the role of dosimetry leading to prohibitive immune organ toxicity or TRT-induced immune suppressive effects preventing the intended priming and propagation of the anti-tumor immune response. If encountered, these barriers could halt or significantly delay the clinical availability of these promising treatments.

Combinations of TRT and immunotherapy have the potential to change patient outcomes in oncology clinics. The therapeutic effectiveness of engaging a patient’s own immune system to eradicate tumor cells at all disease sites has already proven immense. Preclinical and clinical studies are starting to build on the momentum of promising TRT agents. Yet, the radiobiologic and immunologic effects of TRT are not well understood. Therefore, detailed dosimetry studies are required to enable understanding of the effects of absorbed dose, dose rate, dose range, LET, and tissue and cellular distribution of absorbed dose and how these properties affect tumor cells, normal cells, and the TME. It is important for these studies to be performed with different radionuclides and combination therapies in order to define the immuno-radiobiological interface.

Well-designed preclinical veterinary trials with larger subjects, such as companion canines, will aid in clinical translation of TRT-immunotherapy combinations. Ultimately, well designed clinical trials in cancer patients will be essential to widespread TRT-immunotherapy implementation. Development of new training pathways to establish a workforce with TRT-specific expertise, and collaboration between multiple disciplines of medicine and science will be essential to the effective design, conduct, and outcome of these clinical trials. The interface between TRT and immunotherapies proffers abundant potential as a novel approach to treating metastatic cancer, and this is motivating a growing field of scientific and clinical investigation.

## Figures and Tables

**Table 1:** Preclinical Combination Targeted Radionuclide Therapy (TRT)-Immune Checkpoint Inhibitor (ICI) Studies.

Publication	Publication Year	TRT	Target	ICI	Disease Model
Choi et al. [61]	2018	^177^Lu-LLP2A	VLA-4	anti-PD-1/anti-PD-L1 + anti-CTLA-4	B16F10 melanoma
Chen et al. [56]	2019	^177^Lu-EB-RGD	Integrin α_V_β_3_	anti-PD-L1	MC38 colon adenocarcinoma
Rouanet et al. [48]	2020	^131^I-ICF01012	Melanin	anti-CTLA-4, anti-PD-L1, anti-PD-1	B16F10 melanoma
Jiao et al. [62]	2020	^213^Bi-h8C3	Melanin	anti-PD-1	Cloudman S91 melanoma
Malo et al. [63]	2020	^177^Lu- or ^225^Ac-h8C3	Melanin	anti-PD-1	Cloudman S91 melanoma
Dabagian et al. [64]	2021	^211^At-MM4	PARP-1	anti-PD-1	GL261 glioblastoma
Czernin et al. [65]	2021	^225^Ac-PSMA617	Prostate Specific Membrane Antigen (PSMA)	anti-PD-1	RM1-PGLS prostate cancer
Jagodinsky et al. [52]	2021	^90^Y-NM600	Lipid rafts	anti-CTLA-4 + anti-PD-L1	MOC2 head and neck squamous cell carcinoma
Patel et al. [53]	2021	^90^Y-NM600	Lipid rafts	anti-CTLA-4 +/− anti-PD-L1	B78 melanoma, B16 melanoma, 4T1 breast cancer, NXS2 neuroblastoma
Li et al. [66]	2021	^212^Pb-VMT01	Melanocortin 1 receptor	anti-CTLA-4 + anti-PD-1	B16F10 melanoma, YUMM1.7 melanoma
Foster et al. [67]	2021	^177^Lu-Lumi804-αCD11b	CD11b^+^ cells	anti-CTLA-4 + anti-PD-1	GL261 glioma
Guzik et al. [68]	2021	[^177^Lu]Lu-DOTA-folate	Folate receptor	anti-CTLA-4	NF9006 breast cancer
Lejeune et al. [69]	2021	^227^Th-anetumab corixetan	Mesothelin	anti-PD-L1	MC38-hMSLN (human mesothelin) colon adenocarcinoma
Ferreira et al. [70]	2022	^90^Y-GZP	Granzyme B	anti-CTLA-4 + anti-PD-1	MC38 colon adenocarcinoma, CT26 colon carcinoma
Wen et al. [71]	2022	^131^I-anti-PD-L1	Programmed death receptor ligand 1 (PD-L1)	anti-PD-L1	MC38 colon adenocarcinoma, CT26 colon carcinoma
Potluri et al. [72]	2022	^90^Y-NM600	Lipid rafts	anti-CTLA-4 +/− anti-PD-1	TRAMP-C1 prostate cancer, MycCaP prostate cancer
Wen et al. [57]	2022	^177^Lu-DOTA-EB-cRGDfK	Integrin α_V_β_3_	anti-PD-L1	MC38 colon adenocarcinoma, CT26 colon carcinoma

**Table 2:** Clinical Trials of Combination Targeted Radionuclide Therapy (TRT)-Immunotherapy.

Clinical Trial Identifier	Study Start Date	TRT	Target	Immunotherapy	Disease	Status
NCT00438880	Oct 2004	^90^Y ibritumomab tiuxetan	CD20	Agatolimod sodium	Non-Hodgkin lymphoma	Phase I/II; Completed
NCT00450619	Feb 2007	^153^Sm-EDTMP	Bone metastases	Prostate-specific antigen (PSA)/TRICOM vaccine	Metastatic castration resistant prostate cancer (mCRPC)	Phase II; Completed
NCT02463799	Feb 2016	^223^Ra	Bone metastases	Sipuleucel-T	mCRPC	Phase II; Completed
NCT02814669	Sep 2016	^223^Ra	Bone metastases	Atezolizumab	mCRPC	Phase Ib; Completed
NCT03093428	Jun 2017	^223^Ra	Bone metastases	Pembrolizumab	mCRPC	Phase II; Active, not recruiting
NCT03215095	Jul 2017	^131^I	Recombinant human thyroid stimulating hormone (rhTSH) stimulated thyroid	Durvalumab	Thyroid cancer	Phase I; Active, not recruiting
NCT03325816	Nov 2017	^177^Lu-DOTA0-Tyr3-Octreotate	Somatostatin Receptor (SSTR)	Nivolumab	Advanced lung neuroendocrine tumors (NETs)	Phase I; Completed
NCT02914405 (MiNivAN)	May 2018	^131^I-meta-iodobenzylguanidine (MIBG)	Norepinephrine Transporter	Nivolumab	Relapsed/ refractory pediatric neuroblastoma	Phase I; Recruiting
NCT03457948	Aug 2018	^177^Lu-DOTA0-Tyr3-Octreotate	SSTR	Pembrolizumab	Neuroendocrine neoplasm	Phase II; Recruiting
NCT03805594	May 2019	^177^Lu-PSMA-617	Prostate-Specific Membrane Antigen (PSMA)	Pembrolizumab	mCRPC	Phase Ib; Active, not recruiting
NCT03658447 (PRINCE)	Jul 2019	^177^Lu-PSMA-617	PSMA	Pembrolizumab	mCRPC	Phase Ib/II; Active, not recruiting
NCT04071236	Dec 2019	^223^Ra	Bone metastases	Avelumab	mCRPC	Phase I/II; Recruiting
NCT04261855 (GoTHAM)	Mar 2020	^177^Lu-DOTATATE	SSTR	Avelumab	Metastatic Merkel Cell Carcinoma	Phase Ib/II; Recruiting
NCT03996473	Mar 2020	^223^Ra	Bone metastases	Pembrolizumab	Non-small cell lung cancer (NSCLC)	Phase I; Active, not recruiting
NCT04109729	Aug 2020	^223^Ra	Bone metastases	Nivolumab	mCRPC	Phase Ib/II; Recruiting
NCT04946370	Aug 2021	^225^Ac-J591	PSMA	Pembrolizumab	mCRPC	Phase I/II; Recruiting
NCT05583708	Est. Feb 2023	^177^Lu-DOTATATE	SSTR	Pembrolizumab	Merkel Cell Carcinoma	Phase II; Not yet recruiting

## Data Availability

No new data were created or analyzed in this study. Data sharing is not applicable to this article.
